# Quantification of fungal abundance on cultural heritage using real time PCR targeting the β-actin gene

**DOI:** 10.3389/fmicb.2014.00262

**Published:** 2014-05-28

**Authors:** Jörg Ettenauer, Guadalupe Piñar, Hakim Tafer, Katja Sterflinger

**Affiliations:** Department of Biotechnology, Vienna Institute of Biotechnology, University of Natural Resources and Life SciencesVienna, Austria

**Keywords:** fungi, abundance, real-time qPCR, β-actin gene

## Abstract

The traditional methodology used for the identification of microbes colonizing our cultural heritage was the application of cultivation methods and/or microscopy. This approach has many advantages, as living microorganisms may be obtained for physiological investigations. In addition, these techniques allow the quantitative and qualitative assessment of the investigated environment. Quantitative analyses are done by plate count and the determination of abundance by the colony forming unit (CFU). Nevertheless, these techniques have many drawbacks that lead to an underestimation of the cell numbers and do not provide a comprehensive overview of the composition of the inhabiting microbiota. In the last decades, several molecular techniques have been developed enabling many advantages over the cultivation approach. Mainly PCR-based, fingerprinting techniques allow a qualitative detection and identification of the microbiota. In this study, we developed a real time PCR method as a simple, rapid and reliable tool to detect and quantify fungal abundance using the β-actin gene, which is known to appear as a single-copy gene in fungi. To this end, five different indoor thermal insulation materials applied for historical buildings that were previously tested for their bio-susceptibility against various fungi were subjected to qPCR analyses. The obtained results were compared with those obtained from a previous study investigating the bio-susceptibility of the insulation materials using classical cultivation experiments. Both results correlated well, revealing that Perlite plaster was the most suitable insulation material, showing the lowest fungal CFU and qPCR values. In contrast, insulations made of wood showed to be not recommendable from the microbiological point of view. In addition, the potential of qPCR was tested in other materials of cultural heritage, as old parchments, showing to be a suitable method for measuring fungal abundance in these delicate materials.

## Introduction

Our cultural heritage is continuously exposed to the effects of physical, chemical, and biological factors. The latter including biodeterioration caused by microorganisms (Sterflinger and Piñar, 2013). Therefore, the identification of the microorganisms involved in biodeterioration is the first necessary step for understanding the effects of microorganisms on cultural assets. The second step is to elucidate the actual amount, activity and functional stage of these microorganisms and their role in biodeterioration. Finally, the third step is to use the obtained information to develop strategies for the conservation and protection of monuments and art-works (González and Saiz-Jimenez, 2005).

Traditionally, the methodology used for the isolation and identification of microorganisms from different types of materials of our cultural heritage was the application of cultivation methods and/or microscopy. The classical cultivation of microorganisms has offered many advantages, as living microorganisms could be obtained for further physiological investigations. Furthermore, these techniques allowed the quantitative and qualitative assessment of the investigated environment. By using this methodology, quantitative analyses have been done by plate count and the determination of activity by the colony forming unit (CFU), both being analyses based on the growth of microorganisms on selective media. Nevertheless, nowadays these techniques are known to have many drawbacks (e.g., need of considerable sample amounts, great time effort, only a small proportion of cultivable microorganisms present on samples, etc.) that lead to an underestimation of the cell numbers and further do not provide a comprehensive overview of the composition of the inhabiting micro-biota (Ward et al., 1990).

In the last decades, several culture-independent, molecular DNA and phylogenetic techniques have been developed supplying many advantages over the traditional cultivation approach (Amann et al., 1995; Hugenholtz and Pace, 1996; Hugenholtz et al., 1998). Molecular techniques take advantage of the specificity provided by nucleic acid sequences for the identification of microorganisms and their independence of culturing microorganisms. Different, mainly PCR-based, genotyping techniques have been developed and adapted for the fingerprinting of microbial communities on biodeteriorated cultural heritage (Piñar et al., 2001a; Schabereiter-Gurtner et al., 2001; González, 2003; González and Saiz-Jimenez, 2004, 2005; Michaelsen et al., 2006).

These techniques have enabled a reliable study and monitoring of the microbial communities associated with different materials, such as stone, prehistoric caves, wall paintings, oil paintings, historical glasses, paper, parchment, human remains, etc. (Piñar et al., 2001b, 2010, 2011, 2013a,b; Schabereiter-Gurtner et al., 2001, 2002, 2004; Carmona et al., 2006; Michaelsen et al., 2006, 2010, 2013; Bastian et al., 2010; Ettenauer et al.,^2010^, ^2011^; Portillo and González, 2011; López-Miras et al., 2013a,b). To date, molecular techniques are very well established and are complementing to more classical microbiological methods in the study of microorganisms and their role in cultural heritage. The fusion of these two different strategies have delivered complementary results which allow a much better understanding of the identity and diversity of the microorganisms inhabiting our cultural heritage (Laiz et al., 2003, Ettenauer et al., 2010; López-Miras et al., 2013a,b).

In this context, recent investigations have focused on the development of quantitative molecular tools that are broad-coverage, sensitive, and specific. One of these methods is based on real-time quantitative polymerase chain reaction (qPCR), which is a well-known method for microbial detection (Zhang and Fang, 2006). It is based on amplification of specific DNA-regions, monitoring the amplification continuously by using fluorescent dyes, and quantification of the target based on standards. Since the detection is based on DNA, it is not dependent on the cultivability of the microbes. The main advantages of real-time PCR are its quantitative property and high specificity. It is also rapid and easy to perform, after the assay has been set up and validated properly. Quantitative PCR analyses have been widely applied for studying the levels of individual species and assay groups in indoor samples (Haugland et al., 1999, 2004; Meklin et al., 2004; Vesper et al., 2008; Kaarakainen et al., 2009) and in less cases, in building materials (Pietarinen et al., 2008; Pitkäranta et al., 2011). However, few studies have explored the total mycobiota using DNA-based universal community characterization methods, like ribosomal DNA amplicon sequencing or metagenome analysis (Pitkäranta et al., 2008; Tringe et al., 2008; Liu et al., 2012). Nevertheless, it is worth noting that by using universal rRNA primers, it is difficult to calculate single fungal cells in a certain environmental sample. The great variation of the number of rRNA gene clusters in a genome and in a species (Herrera et al., 2009) makes it difficult to estimate the number of fungal individuals.

Therefore, in this study, we have developed a real time PCR method as a simple, rapid and reliable tool to detect and quantify fungi using the β-actin gene. Fungi appear to have a tendency toward a single actin gene copy per haploid genome (Gallwitz and Seidel, 1980; Ng and Abelson, 1980; Mertins and Gallwitz, 1987; Fidel et al., 1988; Voigt and Wostemeyer, 2000), thus enabling a precise quantification of fungal cells. Moreover, comparative sequence analysis of actin information, both at the nucleotide and at the amino acid level, is developing into a highly appreciated tool for long-range phylogenetic studies (Voigt and Wostemeyer, 2000).

To this end, five different indoor thermal insulation materials, based on ecological materials that can be applied for historical buildings, were tested for their bio-susceptibility against various fungi under natural and laboratory conditions (Sterflinger et al., 2013) based on the qPCR targeting the β-actin and the CFU method. In addition, the potential of qPCR for the detection of the β-actin gene was tested in other materials of cultural heritage, as old parchments, which were already investigated and known to be colonized by fungi (Piñar et al., 2011, 2014).

## Materials and methods

### Experimental procedure

Five different indoor insulation materials—bloated Perlite plaster, bloated Perlite board, reed board and loam, wooden soft-board and sprayed cellulose—were evaluated (see Table 1). Therefore, small areas (4 × 4 cm) of the test items (~10 × 10 cm in size) were inoculated with each 1 ml (concentration 10^5^ spores ml^−1^) of 4 different spore solutions from 3 commonly indoors occurring fungi: *Cladosporium cladosporioides* (MA 1610—further named a), *Aspergillus niger* (MA 1615—b) and *Penicillium chrysogenum* (MA 1701—c), and a mixture of all three (d), by plating and spreading the spore solutions with a spatula on the materials surface. These samples were incubated in a climate chamber (Weiss-Klimakammer WKL 100) at 28°C and 90% relative humidity for a period of 6 months. Afterwards, the surface area (to a depth of ~0.5 cm) was removed for cultivation and molecular analysis.

**Table 1:** **Overview of the investigated insulation materials and the fungal strains used for inoculation in this study**.

**Insulation materials**	**Fungal strains (short cut)**
Sprayed cellulose	*Cladosporium cladosporioides* (a)
Bloated Perlite board	*Aspergillus niger* (b)
Bloated Perlite plaster	*Penicillium chrysogenum* (c)
Wooden soft-board	Mixture of all three fungi (d)
Reed board with loam	

Similar investigations were performed with samples of each insulation material collected after 18 months (1st sampling) and 32 months (2nd sampling) after installation from the tentative historical building and were investigated in the laboratory. From the sprayed cellulose, only samples from the 2nd floor could be taken after 32 months.

### DNA extraction

#### DNA extracted from insulation materials

The FastDNA Spin kit for soil from MP Biomedicals (Illkrich, France) was the method of choice for DNA extraction from construction materials (Ettenauer et al., 2012). The kit combines a mechanical lysis, using bead beating, and chemical lysis of the cells. Samples from each material were ground in liquid nitrogen using a sterile mortar and pestle, homogenized in Falcon tubes and, thereof, 100 mg were weighed for DNA extraction.

After DNA extraction, the DNA yield and –purity (A260/A280 ratio) were assessed using the NanoDrop® ND-1000 Spectrophotometer (peqLab Biotechnologie GmbH, Linz, Austria). Afterwards, 7 μl of the extracted DNA were visualized on 1.5% agarose gels by electrophoresis. Further, the DNA was used as template for PCR reactions.

#### DNA extracted from parchment samples

DNA extraction was performed directly from seven parchment samples, dating from the 13th–19th century (Piñar et al., 2014), by using the FastDNA Spin Kit for Soil from MP Biomedicals, as well. The protocol of the manufacturer was slightly modified. About 10–20 mg (2–3 mm^2^) of parchment were placed in the Lysing Matrix E Tubes with the appropriate buffers, and then processed twice in the Fast Prep FP120 Ribolyzer (Thermo Savant; Holbrook, USA) for 30 s at speed 5.5 (m s^−1^), with 5 min intervals on ice. The Lysing Matrix E Tubes were centrifuged at 14.000 × g for 15 min, and the supernatants were transferred to clean 2 ml tubes. The PPS reagent and the binding matrix solution were applied to the supernatant; the suspension was transferred to the provided spin filter and centrifuged at 14.000 × g for 1 min, following the instructions of the manufacturer. DNA was washed twice with 500 ml of the SEWS-M solution and eluted from the binding matrix with 100 ml DNase/Pyrogen Free Water. The DNA crude extracts were further purified prior to PCR amplification with the QIAamp Viral RNA mini kit (Qiagen, Hilden, Germany) with modifications as follows: the washing step was performed twice with 750 μl buffer AW1/AW, rolling the column to allow more contact with the cartridge and leaving the tubes to stand for 2 min with the buffer, prior to centrifugation. The final elution step was repeated twice with 100 μl of 80°C preheated ddH_2_O (Sigma Aldrich, St. Louis, USA) letting the tubes stand for 2 min before centrifugation. After the DNA extraction and purification steps, the concentration and quality of the DNA extracts was assessed using a NanoDrop® ND-1000 Spectrophotometer. The analyses were performed according to the manufacturer's protocol and the extracted DNA was analyzed in duplicate. Finally, the purified DNA was used directly for PCR amplification.

### Quantitative real-time PCR analyses (qPCR)

Quantitative real-time PCR was performed in a BioRad CFX96™ real-time PCR by using the SensiMix Plus™ SYBR-Kit (Bioline). Each 20 μ l reaction contained 10 μ l SensiMix-Plus, 1 μ l 50 mM MgCl_2_ (final conc. 2.5 mM), 0.25 μ l of a 10 pmol/μ l primer solution using the β-actin primers: ACT 512-F (5' ATG TGC AAG GCC GGT TTC GC 3') and ACT 783-R (5' TAC GAG TCC TTC TGG CCC AT 3') (Carbone and Kohn, 1999), 6.5 μ l H_2_O and 2 μ l of DNA template. The amplification conditions were 95°C for 10 min and then 40 cycles of 95°C 15 s, 61°C 20 s and 72°C 15 s. Fluorescence measurements were made at the end of each annealing cycle and an additional measuring point at 80°C (for 1 s) to detect the formation of primer dimers during amplification. A melt curve analysis was made by raising the temperature from 65 to 95°C in 0.5°C steps for 5 s each.

### Standard curves

To enable the quantification of PCR products, standard curves based on threshold cycles were produced by re-amplifying 10-fold dilution series of PCR products from genomic DNA. An aliquot of each dilution (0.035 fg −0.035 ng, equivalent to 1 × 10^2^ − 1 × 10^8^ β-actin copies) in 3 replicates were used as templates in real time PCR.

The DNA standards were generated with the β-actin primers, mentioned above, with the following PCR program: 95°C for 3 min and the 30 cycles of 95°C 30 s, 55°C 30 s, 72°C 30 s and a final elongation step at 72°C for 1 min. PCR was done with a BioRad C1000 thermal cycler using the PCR Master Mix (Promega, Mannheim, Germany) [50 units/ml of TaqDNA Polymerase in a supplied reaction buffer (pH 8.5), 400 μ M dATP, 400 μ M dGTP, 400 μ M dCTP, 400 μ M dTTP, 3 mM MgCl_2_]. Each 100 μ l reaction contained 50 μ l 2x PCR Master Mix, each 1 μ l of forward and reverse primer (stock: 10 pmol/μ l), 43 μ l ultra-pure water and 5 μ l template of genomic DNA of *Aspergillus niger*. The PCR products were cleaned using the QIAquick PCR Purification kit (QIAGEN) and checked for purity on agarose gels and by sequence analysis with database comparison. Concentration of the PCR product was measured spectrophotometrically at 260 nm with a NanoDrop® ND-1000. The resulting PCR products were used to construct standard curves for absolute quantification. The numbers of copies in the standards were calculated using the formula from Le Calvez et al. (2009) and various online-tools, like from the URI Genomics and Sequencing Center (http://cels.uri.edu/gsc/cndna.html). Standard curves were automatically generated by the BioRad Precision Melt Analysis™ software.

### Statistical analysis

A Pearson correlation coefficient was done to compare the results derived from qPCR analyses and those derived from classical cultivation (CFU method) determined in one of our previous studies (Sterflinger et al., 2013). Further ANOVA and pairwise Wilcoxon tests were applied to look at the statistical significance of the differences in fungal abundance between the different materials inspected. All statistics were done with R (R Core Team, 2014).

## Results

### Quantification of the β-actin gene in insulation materials by qPCR analyses

The five selected insulation materials were subjected to qPCR analyses targeting the β-actin gene to quantify the fungal abundance present in such materials. The amount of newly synthesized target-DNA during the ongoing PCR reaction-cycles was measured continually and the emitted fluorescence from SYBR-green binding was detected in real time by the instrument used. The BioRad Precision Melt Analysis™ software allowed the comparison of the β-actin gene copy numbers in the samples with the known concentrations of the standards. The values obtained from each insulation material sample were further extrapolated per ng of extracted DNA from each material, and are shown in Table 2. Representative primer-specific quantification- and standard curves, as well as melt peak charts are shown in Figures S1A–D (Supplementary Data).

**Table 2:** **Results of the qPCR analysis for the insulation materials: calculated copy numbers per ng extracted DNA for the test items and the samples taken from the historical building after 18 and 32 months of installation**.

**Material**	**Climate chamber**					**Historical building**			
	**Inoculated strains—6 months**					**18 months**		**32 months**	
	**a**	**b**	**c**	**d**	**Control**	**1st floor**	**2nd floor**	**1st floor**	**2nd floor**
Sprayed cellulose	55.47 ± 20.62	216.93 ± 25.83	87.32 ± 32.33	*21.68 ± 14.10*	*8.88 ± 5.39*	*1.88 ± 0.95*	*1.84 ± 1.27*	No sample	*3.39 ± 1.06*
Bloated Perlite board	95.26 ± 14.28	167.19 ± 17.04	156.22 ± 109.96	82.83 ± 12.53	*7.98 ± 0.95*	*0.88 ± 0.33*	*2.85 ± 1.99*	*8.75 ± 2.10*	*11.82 ± 10.51*
Bloated Perlite plaster	*5.76 ± 0.14*	*20.38 ± 6.61*	*6.04 ± 5.64*	*27.47 ± 13.18*	*15.93 ± 4.17*	*5.75 ± 5.35*	*4.41 ± 1.21*	*3.85 ± 0.92*	*15.70 ± 1.05*
Wooden soft-board	4174.16 ± 170.36	5480.56 ± 569.09	1092.86 ± 118.93	906.79 ± 139.36	*25.13 ± 11.28*	36.95 ± 12.89	73.10 ± 23.79	*19.94 ± 1.40*	317.33 ± 39.12
Reed board with loam	*29.19 ± 3.78*	35.48 ± 19.46	*25.80 ± 11.55*	*21.59 ± 3.01*	*3.60 ± 1.91*	*19.59 ± 3.04*	91.90 ± 11.65	92.51 ± 22.90	112.34 ± 30.54

Copy number values written in italics are below the detection limit (30.57 copies/ng extracted DNA) of the qPCR assay. (a) MA 1610 Cladosporium cladosporioides, (b) MA 1615 Aspergillus niger, (c) MA 1701 Penicillium chrysogenum, (d) mixture of all three fungal spore suspensions.

The quantitative real time PCR allowed the detection of fungi in all analyzed samples (see Table 2). Compared to the classical cultivation methodology it was possible to detect fungi in the control items (3.6–25.13 copies per ng of extracted DNA from the sample material) using qPCR.

**Figure 1:**
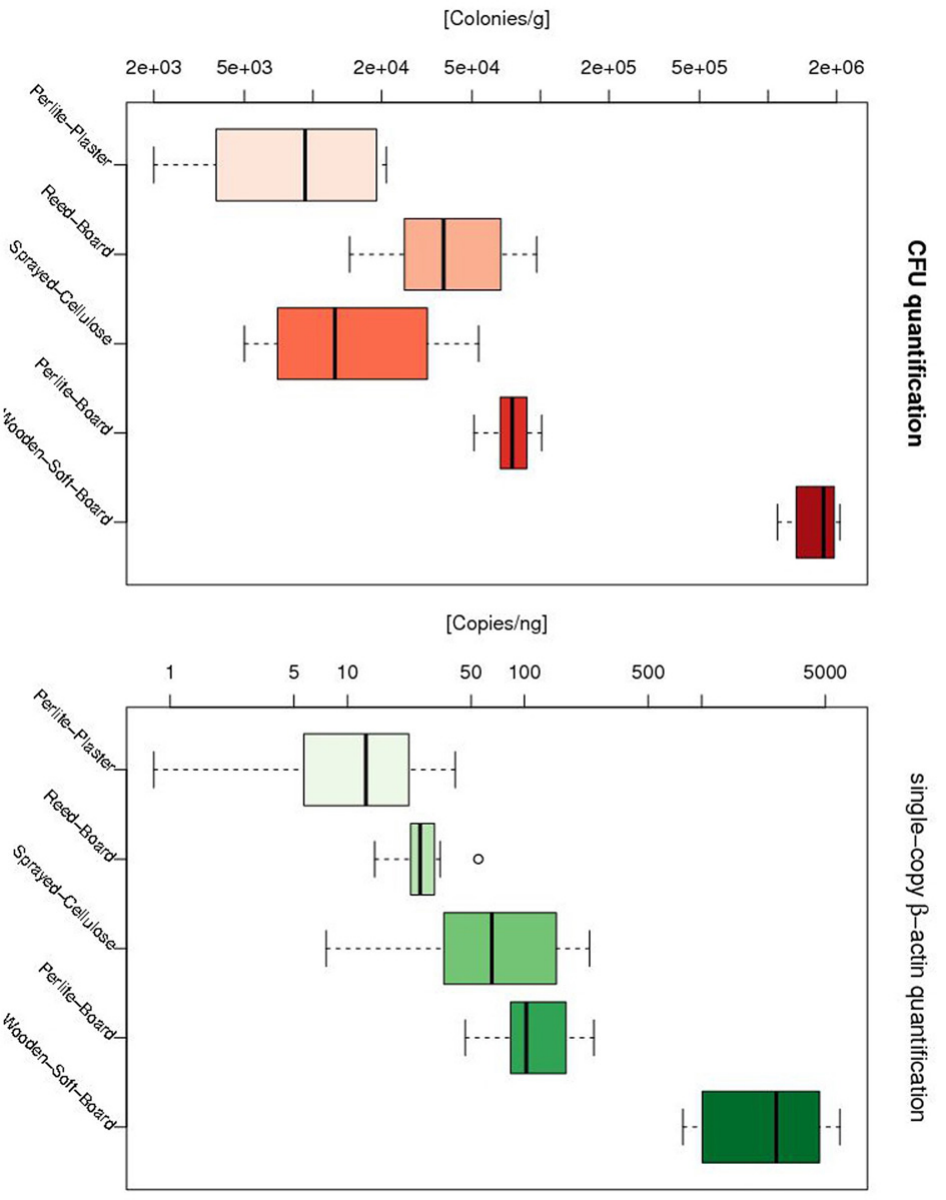
**Distribution of fungal abundance for different growth materials as measured by CFU (l.h.s) and qPCR (r.h.s)**. For both metrics the bloated Perlite plaster and wooden soft-board showed the lowest and highest fungal abundance, respectively. The only inconsistence between CFU and qPCR was seen between the reed board and the sprayed cellulose, where the latter showed a reduced fungal abundance compared to sprayed cellulose based on the qPCR methodology, but a higher fungal abundance based on the CFU approach.

The detected copy numbers per ng of extracted DNA from the different test items ranged from 5.76 to 5480.56. The highest fungal abundance was observed in the wooden soft-board samples (906.79–5480.56 copies/ng), followed by the bloated Perlite board (82.83–167.19 copies/ng) and the sprayed cellulose (21.68–216.93 copies/ng). The lowest β-actin gene numbers were found in the bloated Perlite plaster (5.76–27.47 copies/ng) and the reed board with loam (21.59–35.48 copies/ng) (see Figure 1). The growth differences observed for the different materials were significant (One-Way ANOVA *p*-value: 4.44 × 10–11). In addition, the growth difference between each pair of materials was computed with a Wilcoxon tests, and showed that all but one pair exhibited a *p* < 0.05 (see Table 3). Bloated Perlite plaster and wooden soft-board showed the lowest and the highest fungal abundance, respectively (see Table 2 and Figure 1).

**Table 3:** ***P*-value returned by the Wilcoxon test for the pairwise comparison of fungal abundance for different materials**.

**Material**	**Bloated Perlite board**	**Bloated Perlite plaster**	**Wooden soft-board**	**Reed board with loam**
Sprayed cellulose	0.19	7.17 × 10^−5^	7.40 × 10^−7^	0.0045
Bloated Perlite board		7.40 × 10^−7^	7.40 × 10^−7^	1.48 × 10^−6^
Bloated Perlite plaster			7.40 × 10^−7^	0.0036
Wooden soft-board				7.40 × 10^−7^

Samples obtained from the insulation materials installed in the historical building showed qPCR values ranging from 0.88 to 91.90 copy numbers per ng of DNA after 18 months, and from 3.85 to 317.33 copies/ng after the second sampling (see Table 2). From the wooden soft-board samples, the highest β-actin gene numbers were retrieved (19.94–317.33 copies/ng). The detected copy numbers for nearly all *in situ* samples were 1-2 orders of magnitude lower than the values obtained from the incubated test items. Only the reed board with loam material from the historical building had slightly higher copy numbers, which could be explained by the higher relative humidity in the rooms (19.59–112.34 copies/ng). For the other materials, lower copy numbers–close or below the detection limit of the assay (30.57 copies/ng)—were measured: bloated Perlite board (0.88–11.82 copies/ng), sprayed cellulose (1.84–3.39 copies/ng) and bloated Perlite plaster (3.85–15.7 copies/ng).

### Quantification of the β-actin gene in parchment samples by qPCR analyses

To determine if the quantification of the β-actin gene by qPCR could be used as cellular abundance indicator in other materials of cultural heritage, the DNAs extracted from valuable parchment samples were subjected to qPCR analyses using the same protocol described for insulation materials. The quantitative real time PCR allowed the detection of fungal cells in all parchment samples (see Table 4). The detected β-actin copies were referred to the total amount of DNA extracted from each parchment sample. The resulting values were very similar for all samples and ranged from 168.01–680.09 copies/ng of extracted DNA.

**Table 4:** **Quantification results of the single-copy β-actin gene for the detection of fungal cells on parchment samples by qPCR**.

**Sample**	**Copies/ng extracted DNA**
P1	168.01 ± 5.09
P2	573.84 ± 71.45
P3	337.12 ± 21.15
P5	178.66 ± 1.41
P6	380.55 ± 15.79
P7	680.09 ± 53.23
P8	187.55 ± 23.03

## Discussion

Molecular techniques employing a quantitative real-time PCR have been used for microbial quantification in a variety of environments (Zhang and Fang, 2006; Smith and Osborn, 2009). The method avoids sample limitations and therefore, is particularly suitable for cultural heritage studies. This method has been widely developed for detection of indoor microbes and used for determination of microbes in indoor samples, e.g., house dust, building materials and air (Haugland et al., 1999, 2004; Pietarinen et al., 2008; Vesper et al., 2008; Kaarakainen et al., 2009). However, to date, qPCR has been successfully applied in only very few cultural assets studies. Imperi et al. (2007) used qPCR to investigate the relative abundance of eubacterial and archaeal populations in different wall painting areas suffering from rosy discoloration. Piñar et al., (2010) used qPCR for the specific and sensitive detection and quantification of a *Myxococcus xanthus* strain in a mixed culture used for biological consolidation of ornamental limestone. Martin-Sanchez et al. (2013) developed a qPCR to detect, quantify and monitor *Ochroconis lascauxensis* in the Lascaux Cave in France, being this fungus the principal causal agent of the black stains threatening the Paleolithic paintings of this UNESCO World Heritage Site.

In this study, a qPCR method targeting the β-actin gene was developed for the quantitative assessment of fungi on different insulation materials. The advantage of this method relies in the quantification of a gene that has been proved to appear as a single actin gene copy per haploid genome in fungi (Gallwitz and Seidel, 1980; Ng and Abelson, 1980; Mertins and Gallwitz, 1987; Fidel et al., 1988; Voigt and Wostemeyer, 2000). This fact enables a more precise quantification of the actual amount of fungal cells in an environmental sample than when using universal rRNA primers, due to the great variation of the number of rRNA gene clusters in a genome and among species (Herrera et al., 2009). The results derived from qPCR analysis performed with the different interior insulation systems showed that fungal cells occurred in all samples. The β-actin gene copy number was in nearly all test items higher than the gene copy numbers detected in the samples installed *in situ* in a historical building. Only for the reed board with loam samples, a higher amount of fungal cells was measured after 18 and 32 months of the installation of this material in the building. These findings can be explained by a higher relative humidity in the room during the course of the experiment. The higher humidity further led to increased copy numbers detected on the bloated Perlite plaster and wooden soft-board, whereas the sprayed cellulose and the bloated Perlite board did not show increased cell counts. In these two last materials, the obtained β-actin gene numbers were generally very low and close to, or even below, the limit of detection of the assay. Therefore, the fungal contaminations in the samples can be assumed very low. Furthermore, these results show that the tested insulation materials do not represent optimal growth habitats for fungal colonization.

### Fungal abundance in insulation materials: qPCR vs. cultivation analyses (CFU)

In parallel to the qPCR analyses, classical cultivation analyses were performed with these ecological interior insulation materials ((Sterflinger et al., 2013). Results derived from cultivation analyses proved that actively growing fungi were present in all inoculated test items (see Table S1).

As seen in Figure 1, both methods showed that bloated Perlite plaster and wooden soft-board have the lowest and the highest fungal abundance, respectively. The only inconsistence between CFU and qPCR was seen for the sprayed cellulose. With the qPCR method, the sprayed cellulose showed a higher fungal abundance than the reed board, while with the CFU method the opposite was observed. The CFU and qPCR metrics showed a significant correlation (*p* = 1.123 × 10^−9^, cor = 0.70).

In summary, the results derived from the developed qPCR method correlated well with those obtained in our previous investigations of the different insulation systems using classical cultivation analysis. Taking together the results obtained from both strategies, we conclude that, from the microbiological point of view, the most appropriate interior insulation system is the bloated Perlite plaster. This material achieved the best results: the lowest fungal abundance was detected using the developed qPCR assay and only very few fungal colonies were cultivated from this material. On the contrary, the wooden soft-board system showed to be the most unsuitable material for interior insulation, due to the highest fungal cell numbers detected on this material and the highest CFU values.

### Applicability of the developed qPCR protocol to other materials of cultural heritage

To investigate the application range of the developed qPCR for the detection of cellular abundance in other materials of cultural heritage, the potential of this qPCR was tested on samples retrieved from old parchment manuscripts. These parchment samples were previously investigated due to their heavy damage, consisting of dark purple stains, holes and an unusual powdery consistency of pages. All investigated samples were proved to be colonized by fungi in a previous molecular survey. However, the isolation of fungi from these samples showed negative results (Piñar et al., 2011, 2014). In this study, the results derived from the qPCR analyses showed that this technique was enough sensitive to detect fungi in such valuable artifacts, from which usually a very tiny amount of sample can be obtained for microbiological and/or molecular analyses. This opens the possibility to apply this technique for assessing fungal abundance in other cultural assets, from which always sampling is a limiting step.

In conclusion this study shows that the presented qPCR methodology is a fast, sensitive, direct (without the need of cultivation), and reliable assay for accurately quantifying fungi in different insulation materials and samples of cultural heritage. The approach described can be used to provide new information about fungal abundance in building biological investigations and on microbial habitats on works of art and cultural heritage. Compared to classical cultivation techniques only small sample volumes are necessary which allow a minimal invasive sampling procedure, that is of great importance in the case of object of cultural heritage. Furthermore, the time effort for qPCR analysis is much lower and the drawbacks of cultivation assays, as selectivity and certain detection limits with the use of standard cultivation media, are avoided. Finally, this method enables long range phylogenetic studies at the nucleotide and amino acid level thanks to the sequence information gained from the qPCR, something that is not possible with the traditional CFU method.

## Author contributions

Jörg Ettenauer took samples of the insulation materials in the historical building, did the incubation of test items in the climate chamber, the complete cultivation analysis and the real time experiments. Guadalupe Piñar did the DNA extraction of the parchment samples. Katja Sterflinger designed and managed the project on insulation materials, she is the project leader of the FFG project providing the financial basis of the work and she supervised the lab work. Hakim Tafer did the statistical analysis. Guadalupe Piñar and Jörg Ettenauer wrote the manuscript. Jörg Ettenauer, Guadalupe Piñar, Hakim Tafer, and Katja Sterflinger proof-read the manuscript.

### Conflict of interest statement

The authors declare that the research was conducted in the absence of any commercial or financial relationships that could be construed as a potential conflict of interest.

## Supplementary material

Figure S1 **Representative curves showing the primer-specific quantification curves (A), standard curves (B), melt peak curve (C) and melt curve (D)**.

Table S1 **Summary of the cultivation results from our previous study (Sterflinger et al., 2013) for the insulation materials: calculated colony forming units (CFU) per gram material for the test items and the samples taken from the historical building after 18 and 32 months of installation**.
