# Supplementary material for: Quantification of fungal abundance on cultural heritage using real time PCR targeting the β-actin gene
Source: Front Microbiol. 2014 May 28;5:262. doi: 10.3389/fmicb.2014.00262 (PMC4035567; doi:10.3389/fmicb.2014.00262)
Supplement: Figure S1 — Representative curves showing the primer-specific quantification curves (A), standard curves (B), melt peak curve (C) and melt curve (D). [file Presentation1.ZIP › 87860_Pinar_Supplementary Table 1.docx]

**Supplementary Table 1.** Summary of the cultivation results from our previous study (Sterflinger *et al.,* 2013) for the insulation materials: Calculated colony forming units (CFU) per gram material for the test items and the samples taken from the historical building after 18 and 32 months of installation. Samples that did not show fungal growth are indicated with < 10^2^. (a) MA 1610 *Cladosporium cladosporioides*, (b) MA 1615 *Aspergillus niger*, (c) MA 1701 *Penicillium chrysogenum*, (d) mixture of all three fungal spore suspensions.

| **Material** | **Climate chamber** | | | | | **Historical building** | | | |
| --- | --- | --- | --- | --- | --- | --- | --- | --- | --- |
|  | **Inoculated strains - 6 months** | | | | | **18 months** | | **32 months** | |
|  | **a** | **b** | **c** | **d** | **control** | **1st floor** | **2nd floor** | **1st floor** | **2nd floor** |
| Sprayed Cellulose | 1.70 x10^4^ ± 1.50 x10^3^ | 7.25 x10^3^ ± 2.25 x10^3^ | 4.93 x10^4^ ± 4.25 x10^3^ | 7.00 x10^3^ ± 1.00 x10^3^ | < 10^2^ | < 10^2^ | < 10^2^ | No sample | < 10^2^ |
| Bloated Perlite board | 7.58 x10^4^ ± 5.75 x10^3^ | 9.68 x10^4^ ± 4.25 x10^3^ | 5.70 x10^4^ ± 6.00 x10^3^ | 7.50 x10^4^ ± 2.50 x10^3^ | < 10^2^ | < 10^2^ | < 10^2^ | 2.50 x10^2^ ± 2.50 x10^2^ | < 10^2^ |
| Bloated Perlite plaster | 5.50 x10^3^ ± 1.00 x10^3^ | 2.50 x10^3^ ± 5.00 x10^2^ | 2.10 x10^4^ ± 0 | 1.45 x10^4^ ± 2.50 x10^3^ | < 10^2^ | < 10^2^ | < 10^2^ | < 10^2^ | < 10^2^ |
| Wooden soft-board | 1.97 x10^6^ ± 1.00 x10^5^ | 1.10 x10^6^ ± 7.50 x10^3^ | 1.60 x10^6^ ± 4.50 x10^4^ | 1.95 x10^6^ ± 5.00 x10^4^ | < 10^2^ | 1.50 x10^3^ ± 0 | 1.50 x10^3^ ± 0 | 4.45 x10^4^ ± 2.00 x10^3^ | 4.70 x10^4^ ± 2.00 x10^3^ |
| Reed board with loam | 1.73 x10^4^ ± 2.75 x10^3^ | 9.40 x10^4^ ± 2.00 x10^3^ | 3.18 x10^4^ ± 1.25 x10^3^ | 4.20 x10^4^ ± 0 | < 10^2^ | 1.63 x10^4^ ± 1.25 x10^3^ | 1.20 x10^4^ ± 5.00 x10^2^ | 6.00 x10^3^ ± 5.00 x10^2^ | 6.75 x10^3^ ± 2.50 x10^2^ |
