# Supplementary figures and images for: Quantification of fungal abundance on cultural heritage using real time PCR targeting the β-actin gene
Source: Front Microbiol. 2014 May 28;5:262. doi: 10.3389/fmicb.2014.00262 (PMC4035567; doi:10.3389/fmicb.2014.00262)

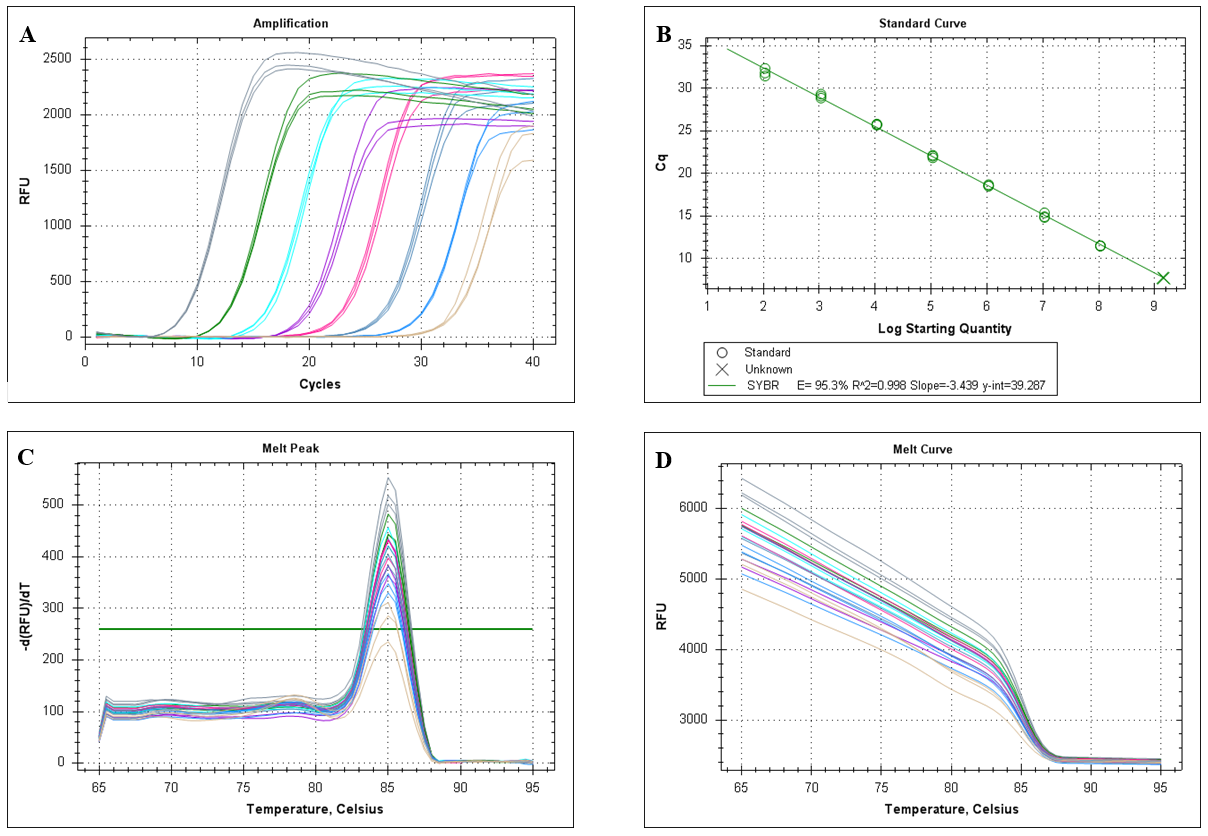

Supplement: Figure S1 — Representative curves showing the primer-specific quantification curves (A), standard curves (B), melt peak curve (C) and melt curve (D). [file Presentation1.ZIP › 87860_Pinar_Figure S1.TIF]
